# Supplementary material for: Poly(GR) and poly(GA) in cerebrospinal fluid as potential biomarkers for C9ORF72-ALS/FTD
Source: Nat Commun. 2022 May 19;13:2799. doi: 10.1038/s41467-022-30387-4 (PMC9119980; doi:10.1038/s41467-022-30387-4)
Supplement: Supplementary file 2 — Supplementary Information [file 41467_2022_30387_MOESM2_ESM.docx]

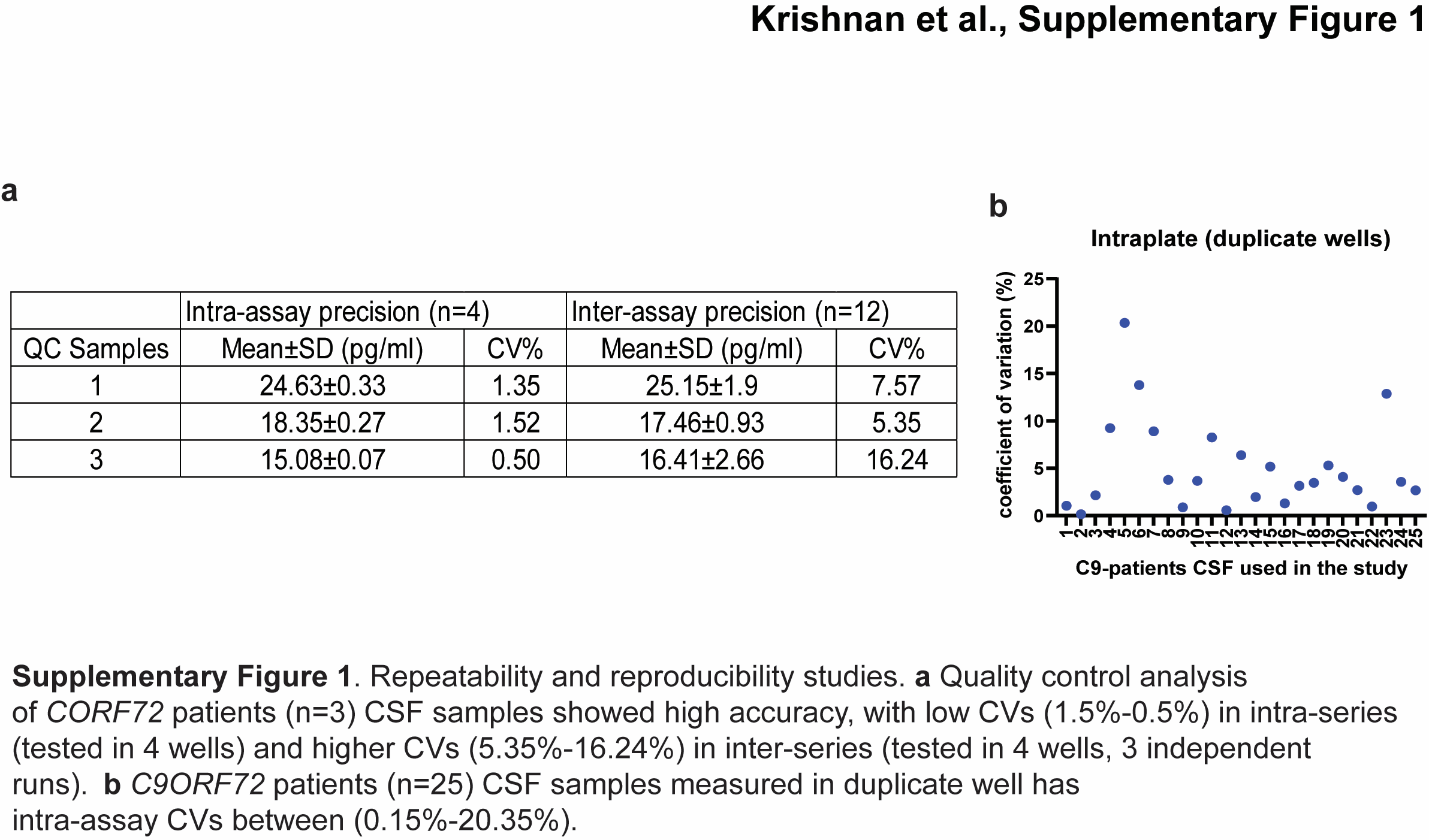


**Supplementary Figure 1.** Repeatability and reproducibility studies. **a** Quality control analysis of patients with *C9ORF72* mutations (n=3) CSF samples showed high accuracy, with low CVs (1.5%-0.5%) in intra-series (tested in 4 wells) and higher CVs (5.35%-16.24%) in inter-series (tested in 4 wells, 3 independent runs). **b** Patients with *C9ORF72* mutations (n=25) CSF samples measured in duplicate has intra-assay CVs between (0.15%-20.35%).


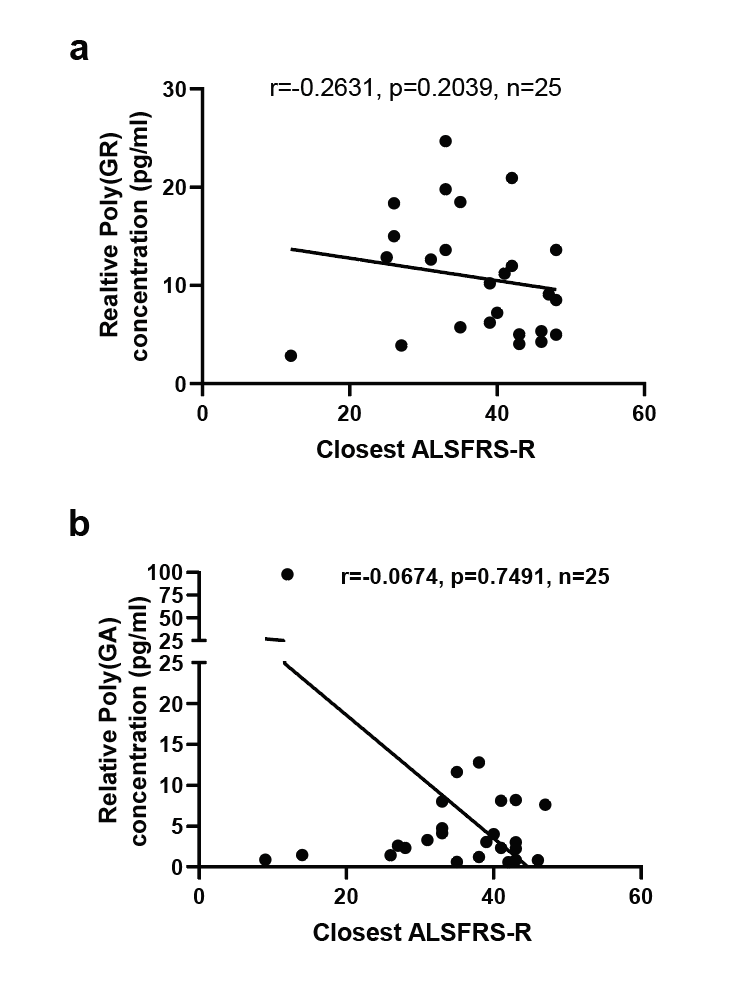


**Supplementary Figure 2.** Poly(GR) (a) and poly(GA) (b) levels in CSF do not correlate with ALSFRS-R scores closest to CSF collection of patients with *C9ORF72* mutations. p values determined by two tailed spearman’s rank correlation analysis. ALS Functional Rating Scale revised (ALSFRS-R)

**Table S1: Clinical characteristics of the participants**

|  | Healthy Control  (n=19)  (Samples used in GR assay) | Non-C9-ALS (n=19)  (Samples used in GR assay) | C9-ALS or ALS-FTD (n=41)  (Samples used in GR assay) | Asymptomatic  (n=10)  (Samples used in GR assay) | C9-ALS or ALS-FTD (n=25)  (Samples used in GA assay) | Asymptomatic  (n=12)  (Samples used in GA assay) |
| --- | --- | --- | --- | --- | --- | --- |
| Average age at time of collection (Years) | 42  (20 to 60) | 57.7  (32.01 to 72.9) | 60  (43 to 73.1) | 48  (29 to 63.3) | 59  (43.1 to 70.9) | 45  (29 to 63.3) |
| Gender (F/M) | (8/11) | (5/14) | (13/28) | (7/3) | (7/18) | (10/2) |
| Average Disease Duration  (Years from symptom onset to sample collection) | Not applicable | 2.02  (0.2 to 4.4) | 2.0  (0.49 to 7.9) | Not applicable | 2.3  (0.49 to 7.9) | Not applicable |
| Average ALSFRS-R at time of first screening | Not applicable | 38.6  (48 to 32) | 34.8  (48 to 9) | 47  (44 to 48) | 34  (47 to 9) | 47  (44 to 48) |
| Onset location  Bulbar  Limb  No data | Not applicable | 1  14  4 | 11  12  18 | Not applicable | 4  7  14 | Not applicable |

**Table S2: Longitudinal Samples details used in Figure 4A**

|  | Sex | Age at onset |
| --- | --- | --- |
| Pre symptomatic |  |  |
| LP00020  LP00157 | M  F | NA  NA |
| Symptomatic |  |  |
| LP00099  LP00192* | M  M | 62  59 |

*Still alive, disease duration to date
