# Supplementary material for: Poly(GR) and poly(GA) in cerebrospinal fluid as potential biomarkers for C9ORF72-ALS/FTD
Source: Nat Commun. 2022 May 19;13:2799. doi: 10.1038/s41467-022-30387-4 (PMC9119980; doi:10.1038/s41467-022-30387-4)
Supplement: Supplementary file 3 — Reporting Summary [file 41467_2022_30387_MOESM3_ESM.pdf]

nature portfolio

Corresponding author(s): Fen-Biao Gao, Mark W. Kankel

Last updated by author(s): Apr 12, 2022

Reporting Summary

Nature Portfolio wishes to improve the reproducibility of the work that we publish. This form provides structure for consistency and transparency in reporting. For further information on Nature Portfolio policies, see our Editorial Policies and the Editorial Policy Checklist.

Statistics

For all statistical analyses, confirm that the following items are present in the figure legend, table legend, main text, or Methods section.

n/a	Confirmed	
☐	☒	The exact sample size (*n*) for each experimental group/condition, given as a discrete number and unit of measurement
☐	☒	A statement on whether measurements were taken from distinct samples or whether the same sample was measured repeatedly
☐	☒	The statistical test(s) used AND whether they are one- or two-sided<br>*Only common tests should be described solely by name; describe more complex techniques in the Methods section.*
☐	☒	A description of all covariates tested
☐	☒	A description of any assumptions or corrections, such as tests of normality and adjustment for multiple comparisons
☐	☒	A full description of the statistical parameters including central tendency (e.g. means) or other basic estimates (e.g. regression coefficient) AND variation (e.g. standard deviation) or associated estimates of uncertainty (e.g. confidence intervals)
☐	☒	For null hypothesis testing, the test statistic (e.g. *F*, *t*, *r*) with confidence intervals, effect sizes, degrees of freedom and *P* value noted<br>*Give P values as exact values whenever suitable.*
☒	☐	For Bayesian analysis, information on the choice of priors and Markov chain Monte Carlo settings
☒	☐	For hierarchical and complex designs, identification of the appropriate level for tests and full reporting of outcomes
☐	☒	Estimates of effect sizes (e.g. Cohen's *d*, Pearson's *r*), indicating how they were calculated

Our web collection on statistics for biologists contains articles on many of the points above.

Software and code

Policy information about availability of computer code

Data collection	Meso Scale Discovery Workbench, version 4 was used to collect poly(GR) signals. Meso Scale Discovery Workbench, Version 4 was used to collect poly(GA) and poly(GP) signals.
Data analysis	Statistical analyzes were performed using GraphPad Prism Version9. Statistical analysis for poly(GA) and poly(GP) were performed using GraphPad Prism Version 7.

For manuscripts utilizing custom algorithms or software that are central to the research but not yet described in published literature, software must be made available to editors and reviewers. We strongly encourage code deposition in a community repository (e.g. GitHub). See the Nature Portfolio guidelines for submitting code & software for further information.

Data

Policy information about availability of data

All manuscripts must include a data availability statement. This statement should provide the following information, where applicable:

- Accession codes, unique identifiers, or web links for publicly available datasets
- A description of any restrictions on data availability
- For clinical datasets or third party data, please ensure that the statement adheres to our policy

Source data underlying Figs. 1–4 and Supplementary Fig. 1 are available as a Source Data file.

Field-specific reporting

Please select the one below that is the best fit for your research. If you are not sure, read the appropriate sections before making your selection.

- [x] Life sciences
- [ ] Behavioural & social sciences
- [ ] Ecological, evolutionary & environmental sciences

For a reference copy of the document with all sections, see nature.com/documents/nr-reporting-summary-flat.pdf

Life sciences study design

All studies must disclose on these points even when the disclosure is negative.

Sample size	We obtained as many CSF samples as we could for this study.
Data exclusions	CSF Samples with GR/GA signals below the limit of detection was not included in the study
Replication	Synthetic GR8 peptide and 3 quality control samples was used to test the assay reproducibility and all replications were successful, 4 quality control samples were included in all the GA assay plates. Poly(GA) and poly(GP) assay reproducibility was assessed by testing the standard curve, three diluent QC samples and an endogenous QC samples across six assay runs over three days, performed by two different operators. Both assays met the per-specified performance acceptability criteria. During CSF testing, two replicates of each CSF sample were tested within the same assay plate and run.
Randomization	Does not apply to this study as Patient samples are limited
Blinding	All the measurements were done in a blinded manner

Reporting for specific materials, systems and methods

We require information from authors about some types of materials, experimental systems and methods used in many studies. Here, indicate whether each material, system or method listed is relevant to your study. If you are not sure if a list item applies to your research, read the appropriate section before selecting a response.

Materials & experimental systems

n/a	Involved in the study	
☐	☒	Antibodies
☒	☐	Eukaryotic cell lines
☒	☐	Palaeontology and archaeology
☒	☐	Animals and other organisms
☐	☒	Human research participants
☒	☐	Clinical data
☒	☐	Dual use research of concern

Methods

n/a	Involved in the study	
☒	☐	ChIP-seq
☒	☐	Flow cytometry
☒	☐	MRI-based neuroimaging

Antibodies

Antibodies used	Custom made GR antibodies produced in Rabbit against synthtic (GR)8 peptie (Covance)
Validation	GR antibodies validation reported earlier in Choi SY et al., Nat Neurosci. 22, 851-862 (2019); GA antibodies validation reported in 26. Nguyen, L. et al., Neuron 105, 645-662.e11 (2020).

Human research participants

Policy information about studies involving human research participants

Population characteristics	CSF samples were collected by other groups and were all publsihed before. Thius study did not directly involve human research participants.
Recruitment	*Describe how participants were recruited. Outline any potential self-selection bias or other biases that may be present and how these are likely to impact results.*
Ethics oversight	*Identify the organization(s) that approved the study protocol.*

Note that full information on the approval of the study protocol must also be provided in the manuscript.
